# A general framework for governing marketed AI/ML medical devices

**DOI:** 10.1038/s41746-025-01717-9

**Published:** 2025-05-31

**Authors:** Boris Babic, I. Glenn Cohen, Ariel Dora Stern, Yiwen Li, Melissa Ouellet

**Affiliations:** 1 University of Hong Kong, Hong Kong, SAR China; 2 University of Toronto, Toronto, ON Canada; 3 The Petrie-Flom Center for Health Law Policy, Biotechnology, and Bioethics at Harvard Law School, The Project on Precision Medicine, Artificial Intelligence, and the Law (PMAIL), Cambridge, MA USA; 4 Harvard Law School, Cambridge, MA USA; 5 Hasso Plattner Institute, University of Potsdam, Potsdam, Germany

**Keywords:** Diseases, Business, Technology, Ethics, Government, Law

## Abstract

This project represents the first systematic assessment of the US Food and Drug Administration’s postmarket surveillance of legally marketed artificial intelligence and machine learning based medical devices. We focus on the Manufacturer and User Facility Device Experience database—the FDA’s central tool for tracking the safety of marketed AI/ML devices. In particular, we evaluate the data pertaining to adverse events associated with approximately 950 medical devices incorporating AI/ML functions for devices approved between 2010 through 2023, and we find that the existing system is insufficient for properly assessing the safety and effectiveness of AI/ML devices. In particular, we make three contributions: (1) characterize the adverse event reports for such devices, (2) examine the ways in which the existing FDA adverse reporting system for medical devices falls short, and (3) suggest changes FDA might consider in its approach to adverse event reporting for devices incorporating AI/ML functions.

## Introduction

There is substantial discussion on whether the U.S. Food and Drug Administration (FDA)’s current regulatory regime of medical devices that incorporate Artificial Intelligence and Machine Learning functions (AI/ML devices) is adequate^1–3^. The fact that the FDA’s newly-established Digital Health Advisory Committee dedicated its inaugural meeting to discussing “total product lifecycle considerations for Generative Artificial Intelligence (AI)-enabled devices” is a testament to the prominence and priority of the topic among regulators^4^. Many critiques and concerns associated with AI devices focus on representativeness, diversity, and bias in the training and validation data of a particular AI/ML device^1^. In this study, we focus on a different dataset, which has not yet been sufficiently scrutinized—namely, the FDA’s system for reporting on adverse events associated with medical devices, which is known as the Manufacturer and User Facility Device Experience (MAUDE) database.

We focus on postmarket surveillance of legally marketed medical devices and how existing regulatory practice interacts with the rapid emergence of AI/ML devices. As of August 2024, the FDA had authorized 950 AI/ML devices^5^. While FDA has a long-standing and comprehensive system for reporting adverse events associated with all medical devices into its MAUDE database, it is unclear how well this existing system works for capturing the kinds of issues and problems that are especially likely to arise from AI/ML devices.

We examine FDA’s MAUDE database from 2010 to 2023. In particular, we evaluate the data pertaining to adverse events associated with medical devices incorporating AI/ML functions for devices approved between 2010 through 2023. We make three contributions: (1) we characterize the adverse event reports for such devices, (2) we examine the ways in which the existing FDA adverse reporting system for medical devices falls short, and (3) we suggest changes FDA might consider in its approach to adverse event reporting for devices incorporating AI/ML functions in the service of more effective post-market surveillance and public health.

This project proceeds as follows. We first describe the regulatory background of the FDA’s medical device reporting (MDR) system and the MAUDE database as well as the data collected for this study and the central results, including the limitations of the MAUDE database and some fundamental problems of a regulatory regime centered around postmarket surveillance. In the Discussion, we explore two possible avenues for improving the current regime: first, how to improve the system of postmarket surveillance so as to better track problems that are particularly salient to AI/ML devices and, second, whether to move to a different regulatory regime altogether. In Methods we describe how we obtained our final dataset, namely, using FDA’s downloadable 510(k) files and NyquistAI’s database, thereby arriving at a final dataset which comprises 823 unique FDA cleared AI/ML devices corresponding to a total of 943 subsequent adverse events reported (MDRs for short) between 2010 and 2023.

In the United States, modern medical device regulation began with the 1976 Medical Device Amendments (MDA) to the 1938 Federal Food, Drug and Cosmetic Act (FDCA)^6^. The FDA’s medical device reporting (MDR) system is one of the central postmarket surveillance tools for managing medical device related adverse events and ensuring the ongoing safety and efficacy of products once they are authorized for marketing and used in patients.

The core reporting requirements for medical device adverse events are laid out in the Medical Device Reporting Regulation (21 CFR, Part 803), which was published on December 11, 1995 (60 FR 63578), and is authorized by Section 519 of the FDCA (21 CFR, Part 803 and 60 FR 63578). The MDR system requires manufacturers, user facilities, and importers of legally marketed medical devices to submit reports of certain adverse events involving their medical devices (21 CFR 803.10(a)-(c)). The FDA refers to these as “MDR reportable events.”^7^ MDR reportable events for device manufacturers include reports of death, serious injury, or device malfunction. A malfunction is reportable if it would be likely to cause death or serious injury if the malfunction were to recur (21 CFR 803.3 and 21 CFR 803.50). MDR reportable events include 30-day reports and 5-day reports. 5-day reports are generally only required when a reportable event requires remedial action to prevent an unreasonable risk of substantial harm to the public health (21 CFR 803.10(c)). Any of the following issues can be a factor in an MDR reportable event: failure, malfunction, improper or inadequate design, manufacture, labeling, or user error (21 CFR 803.3).

The current regulatory system for postmarket surveillance has emerged out of an infrastructure which was initially developed for hardware-only medical devices (such as stents and hip implants), and eventually evolved to regulate software features embedded in medical devices (such as the software powering large pieces of digital radiology equipment) as well as software products that themselves meet the legal definition of a medical device according to the FDA (such as simple diagnostic algorithms)^8^. However, AI/ML devices present unique and different challenges relative to existing devices–both traditional and digital. For example, AI/ML systems may be trained on a set of data from one population but used in a population with very different characteristics and perform poorly in that new population^9–11^. Likewise, the distribution of the population characteristics on which they are deployed may simply shift over time. These concepts are referred to as “concept drift” (which refers to the change in the relationship between the population characteristics and the target variable under study) and “covariate shift” (which refers to the change in the distribution of the population characteristics alone) and we address them and their relevance for error reporting in more detail below^9–13^. An AI/ML device may also not be robust and it can provide very different outcomes for similarly situated patients^14^. Further, it may perform particularly poorly with respect to specific or rare disorders^15^. Finally, the device may reflect bias for certain racial or other groups but perform well across the full population (an instance of the well-known Simpson’s Paradox)^16^. While all of these are salient and well recognized problems that can plague AI/ML systems, none of these issues can be well-categorized using the typical constructs that FDA uses for reporting a device “malfunction.” We designed this study, in part, to determine whether the existing reporting system is fit-for-purpose for monitoring adverse events associated with medical devices incorporating AI/ML functions. In a related study, Correia et al. examine the MAUDE database to evaluate the sources of problems contributing to adverse event reports. Looking at data from 2015 to 2021, they find that user errors and the way that AI/ML devices are applied in practice are particularly likely to cause harm. In this study, we consider a wider dataset, and we attempt to offer concrete lessons on what makes AI/ML device problems unique (as opposed to traditional, hardware-only medical devices) and what specifically the FDA can do to better mitigate those problems^17^.

## Results

**Fig. 1:**
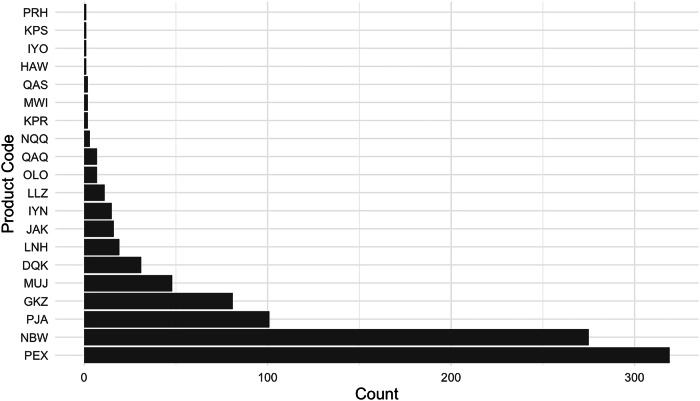
Distribution of adverse events by product code. This figure presents the number of reported adverse events (MDRs) linked to each of the 20 product codes identified in our merged dataset of FDA-cleared AI/ML-enabled devices and adverse event reports. The product code *PEX* corresponds to Biomerieux’s Mass Spectrometry Microbial Identification System, and *NBW* refers to Dario’s Glucose Monitoring System.

As described further in Methods, we collected data from the FDA’s medical device adverse event reporting database (the “Manufacturer and User Facility Device Experience” or MAUDE database). Our final dataset (which we have made publicly available, together with all the associated code, through the links at the end of this manuscript) comprises 823 unique 510(k)-cleared devices that could be linked to a total of 943 subsequent adverse events reported (MDRs for short) between 2010 and 2023. The vast majority of AI/ML device MDRs come from two products (See Fig. 1). The first is Biomerieux’s Mass Spectrometry Microbial Identification System (product code PEX), an automated mass spectrometer system utilizing matrix-assisted laser technology for the identification of microorganisms cultured from human specimens. This system is designed to provide rapid and accurate identification of a wide range of microorganisms, and to assist healthcare professionals in diagnosing infections and guiding appropriate microbiology treatment plans. The second is DarioHealth’s Dario Blood Glucose Monitoring System (product code NBW), a direct-to-consumer software product which produces blood glucose level readings through its smart app.

A first surprising finding is that there is an extremely high concentration of MDRs in such a small number of the AI/ML devices. While one also observes concentration in MDRs for medical devices *without* AI/ML functions, the concentration is not as extreme. In Supplementary Information, we provide a figure (Supplementary Fig. 1) comparing the market concentration, so to speak, of adverse events in AI/ML devices vs. non AI/ML devices, as well as an associated figure (Supplementary Fig. 2) comparing event types across AI/ML devices and non AI/ML devices. We find that more than 98% of adverse events occurring within AI/ML devices are borne by less than five devices. For non AI/ML devices, the corresponding figure is about 85%. Meanwhile, with respect to the “event type,” 90.88% of AI/ML device reports are reported as malfunctions, whereas for non AI/ML devices, the corresponding figure is 77.05%. Under both measures, therefore, the concentration for AI/ML devices is particularly extreme.

Most MDRs associated with the Mass Spectrometry Microbial Identification System (PEX) are reported as misidentifications of microorganisms. Many of these issues appear to stem from limitations in the system’s knowledge base, which identifies microorganisms by comparing test results to known profiles. It is difficult to understand the true severity of these problems from the available data. Misidentification of microorganisms can be very dangerous, even life threatening. But from the information provided in regulatory databases, it is hard to decipher whether the events reported constitute this level of severity and, if they do, whether the AI/ML device is responsible for the event. This is emblematic of more general issues, we discuss below, as to why the current reporting structure is not fit for purpose for evaluating AI/ML devices.

Meanwhile, for DarioHealth, the main issues reported are associated with incorrect blood glucose level readings, at least some of which could be interpreted as false positives. We do not intend to single out these products as poorly performing—while they are overrepresented in the database, this does not allow for any general conclusion about their quality. Indeed, in the absence of data about the overall frequency of a device’s use and clarity on the salience of problems with the device relative to other devices, it is difficult to speculate about a device’s relative performance or safety. It may even be the case that these products are overrepresented because the relevant manufacturers are particularly diligent about their reporting duties and/or quality control.

In any case, we highlight these examples to illustrate the general character of the adverse event reports that are most represented among AI/ML device MDRs, which is characterized by the kinds of malfunctions and product issues that would traditionally be important in non-AI/ML devices. These types of malfunctions are not always salient for AI/ML devices, hence the value of the reports is limited in the context, and the burden imposed on manufacturers may be unnecessary and/or unequal. We explain the shortcoming of the current system in more detail below.

There are several important limitations of the current MDR system which make it suboptimal for tracking, understanding, and correcting safety issues that arise with AI/ML medical devices. Consistent with the patterns described above, we now further describe the main issues identified in the data.

### Missing data

A significant concern with the current state of the MAUDE database is simply the sheer extent of missing data within MDRs–and this is even before one considers selection issues associated with whether adverse events are reported at all. The problem of missing data for FDA cleared AI/ML devices has been raised elsewhere^18^, but to our knowledge no one has systematically investigated the missing data in the MAUDE reports of AI/ML devices.

**Fig. 2:**
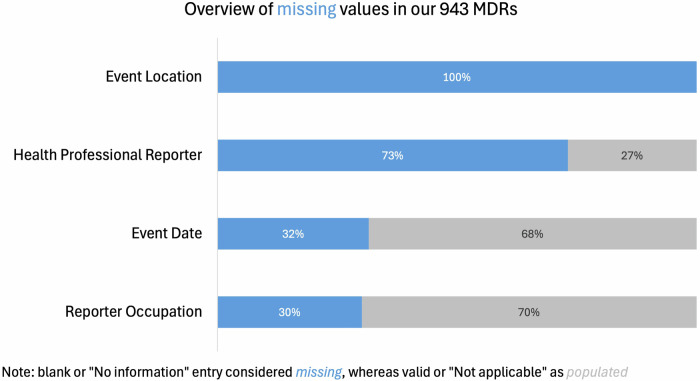
Proportion of missing values across key fields in medical device reports. This chart illustrates the extent of missing information across four key fields in a total of 943 medical device reports (MDRs) associated with AI/ML-enabled devices. “Missing” includes blank entries or those marked as “No information,” whereas entries marked “Not applicable” were treated as populated.

Missing data entries within the formal MDRs make it difficult to study AI/ML medical device safety effectively from a quantitative perspective. Figure 2, below, presents the extent of missing information for four important indicators in the MAUDE database. Note that each of the color coded categories below represent different ways in which data can be missing. While they are coded differently—the result is the same.

In the analysis sample of 943 adverse event reports, data completeness varied significantly across key variables. We consider blank or “No Information” entries as missing data, and we consider completed or “Not applicable” entries as populated data. Event Location is entirely missing for all MDRs in the sample (*n* = 369 entries are blank, and *n* = 574 are marked as “No information”). Similarly, 73% of the reports lack information about whether the reporter was a health professional (*n* = 509 blank, *n* = 101 “No information”). Event Date is missing in 32% of the reports (*n* = 298), while Reporter Occupation is absent in 30% of cases (*n* = 283 blank, *n* = 1 “Not applicable”). The limited data availability regarding key contextual features of adverse events highlights potential gaps in the reporting process. Yet such information is especially important for AI/MLmedical devices, whose performance is known to be contextually sensitive—such devices’s ability to perform as intended can deteriorate significantly in a different sub-population or when used by different parties.

Most importantly, the extent of missing data was significantly higher in the AI/ML sample as compared to the frequency of missing MDR information for other medical devices. For instance, information about whether the reporter was a health professional was missing 73% of the time in the AI/ML sample, but only 43% in the overall sample. Similarly, Event Date and Reporter Occupation were more likely to be missing in the AI/ML sample (32% vs 21.9% and 30% vs 12.7%, respectively). Event Location was entirely missing (100%) in the AI/ML sample, compared to 90.1% in the general device sample.

Above all, these data deficiencies create difficulties for policymakers, scholars, and manufacturers in accessing and investigating the specific causes of adverse events related to AI/ML devices. Especially for reports submitted by non-health professionals, the absence of key event details such as timing, location, and reporter information would require manufacturers to spend more time on follow-ups to gather additional details. Hence, the overall result is an incomplete picture of the safety issues that the database is intended to capture.

Indeed, a closer look at the MDRs reveals that in many cases, even after self-reported extensive follow-up efforts, manufacturers struggled to obtain more information. For example, in an NBW device report (i.e., DarioHealth’s Dario Blood Glucose Monitoring System) where both the event location and reporter information were missing, the manufacturer DarioHealth mentioned that “the user refused to troubleshoot with Dario’s representatives. There is not enough information available regarding Dario meters to investigate”^19^. In another report with significant information gaps, it was similarly noted that, “multiple attempts to follow up with the user were made, however, no response has been received to date”^20^. With frequent missing information in the reports, it becomes challenging for manufacturers to fully understand the context of device issues and malfunctions, thereby limiting their ability to conduct thorough device assessments and provide resolutions. Moreover, the inability to determine the specifics of device failures, particularly for medical events involving serious injury or death, complicates the assignment of responsibility. These data gaps challenge the ability of both manufacturers and regulators to validate the credibility of the reports and to determine the reporter’s level of expertise.

In light of these challenges, it is necessary to reinforce the standardization and completeness of the current MDR data collection process. This is of course true more generally, but in the relatively new context of AI/ML devices, where little regulatory history exists, it is likely to be particularly valuable for quality improvement efforts and, therefore, protecting the public’s health. Furthermore, because there are unique concerns related to transferability (how a model applies across contexts), domain adaptation (how the model adapts to new contexts), and cross-dataset evaluation of AI/ML models (the model’s robustness across different datasets), understanding performance of such algorithms *in context* is especially important. Such improvements in data collection would not only help manufacturers obtain more complete event information but would also facilitate effective quantitative analysis of the reports database and enable regulators to implement corrective measures more efficiently.

### Inadequate event classification

The current database contains a significant proportion of inadequate event classifications—events that are reported a certain way in the database, but when one reads the qualitative description, the recorded information does not seem to match the report, reflecting a disconnect between the actual challenges arising in practice and the categorical constraints of the current reporting system. According to the latest MDR guidelines, all submitted MDRs are classified into three categories: Malfunction (M), Injury (I), and Death (D), which are recorded under Event Type in the MAUDE database.

As seen in Table 1, the majority (91%) of device reports are categorized as ‘Malfunction’, while only two events (0.21%) are classified as ‘Death’. Both ‘Death’ events came from DarioHealth’s Dario Blood Glucose Monitoring System (product code NBW). The FDA stipulates that an MDR should only be classified as a death when the reporter believes that the patient’s *cause* of death was or may have been attributed to the device. However, in both death-classified reports from DarioHealth’s glucose monitoring system, the death was reported *not* to be related to the device. In one such report, on November 30, 2018, the spouse of a Dario Blood device user called to report her husband’s death, but it was then subsequently clarified that the death was unrelated to the device^21^. Similarly, on February 6, 2019, another Dario Blood user’s husband contacted Dario to report the user’s death; however, she noted that the user had many health complications and there was no indication the device was involved^22^. As such, both “deaths” appear to be inaccurate classifications in the data, as we cannot conclude on this basis that the deaths are causally attributable to the device.

**Table 1:** Breakdown of adverse event reports by product code and event type

Product Codes	M	IN	D	Total
DQK	30	1	0	31
GKZ	80	1	0	81
HAW	0	1	0	1
IYN	14	1	0	15
IYO	1	0	0	1
JAK	15	1	0	16
KPR	2	0	0	2
KPS	0	1	0	1
LLZ	11	0	0	11
LNH	3	16	0	19
MUJ	43	5	0	48
**MWI**	2	0	0	2
**NBW**	231	42	0	275
NQQ	1	2	0	3
OLO	6	1	0	7
PEX	316	3	0	319
PJA	94	7	0	101
PRH	0	1	0	1
QAQ	7	0	0	7
QAS	1	1	0	2
***n*** **%**	857 90.88	84 8.91	2 0.21	943 100

This table presents the number of adverse event reports in our dataset (*n* = 943), disaggregated by product code and event type. Event types include Malfunction (M), Injury (IN), and Death (D). The table highlights how reports are distributed across both dimensions, with totals shown per product code and per event type.

While extreme, these cases illustrate the difficulty of accurately reporting adverse events for medical AI/ML devices. Doing so requires sorting out thorny issues of causality because death *while using a device* is clearly quite different from *death due to the device* (and its malfunctioning). Moreover, even if death could be attributed to use of the device, that would still be different from death due to the device’s *AI/ML system*. Thus, given how far removed the event is from the AI/ML functionality, there are few if any conclusions that one can reliably draw about AI/ML device safety on the basis of what is reported.

Similar and likely far more common inadequate event classification issues are also evident in non-death adverse event reports. On January 18, 2019, a patient performed a Heart Flow Analysis (product code PJA) in a hospital and received a negative result. Later, the patient experienced serious chest pain and received a CT scan, during which the previous diagnosis result was found to be a false negative. The patient was then urgently referred for cardiac catheterization. This event was reported as an Injury. However, subsequent investigations by Heart Flow revealed that the false negative result was due to an analyst’s mistake rather than a problem with the device software itself^23^. The upshot here is the same as above.

Recording the event as an “Injury” does not seem to be accurate, as the cause of injury was not the device itself (but rather, in this case, an analyst’s mistake). While such misattributions may also occur for other types of medical devices, the complexity of AI/ML product use opens up a new dimension for such user errors. What is particularly interesting is that while one might have predicted that the ambiguity of attributing responsibility could lead to *under* reporting, it appears to have led to over reporting (i.e., Heart Flow reporting a false negative which is not due to a malfunction of its device). Yet there is very little oversight of reporting activities, so the best that regulators and researchers can do is take reports at face value. It would be very useful to have an independent investigation of serious injuries or deaths before the reports are filed so that we can conclude with more confidence whether the device or its AI/ML functionality was related to the event. In any case, such clear retroactive discrepancies suggest that the MDR system might be tagging device events based on patient outcomes rather than outcomes directly caused or potentially caused by the devices.

Relying on the event type to determine the nature of events might lead to the misperception that the medical AI/ML device caused a death, when in fact, that would require a much more thorough investigation. Such challenges also occur in the context of traditional devices, but in the case of AI/ML devices, they are particularly salient: AI/ML is unique because the error can be hard to detect, and its source even harder to identify. Moreover, whether or not something is an AI/ML error to begin with can be challenging to determine. These facets, together with the novelty of the AI/ML products themselves and the lack of familiarity by medical professionals, may lead to more user errors and/or to a specific type of inadequate classification of MDRs, due to lack of product knowledge. The current system, therefore, likely generates an overall inaccurate picture of the safety of these devices.

The preceding event classification problems revolve around some explicitly inadequate or inaccurate statements in a report (for example, an injury is attributed to a device when in reality the injury occurred due to an analyst’s error). But the current environment is also susceptible to inadequate event reporting due to, what we might call, errors of omission; however, the extent of that problem is difficult to estimate. To explain, we provide a hypothetical example. Suppose that an AI/ML system is incorporated into a certain piece of clinical decision support software. And suppose further the entire product is deployed in a triage environment. Now suppose the product is not working well, and is not useful – in the sense that attending physicians do not find it helpful for optimizing their workflow, or even improving their workflow, relative to their performance without the product. In this case, the attending physicians may simply ignore that product. But that does not mean they will report it to the manufacturer as a malfunction, and it likewise does not mean that the manufacturer will file an official report. Indeed, lack of usability is not ordinarily thought of as a reportable issue. As a result, this example depicts a situation where the environment does not incentivize anyone to create a report that would allow researchers and regulators to become aware of a real problem. This is the medical analogue to a phenomenon that has been commonly observed in the context of criminal recidivism prediction—namely, the fact that we can only observe arrest rates, but not offense rates (i.e., we do not observe committed crimes for which the offenders are not caught)^24,25^. Likewise, we do not observe problems that are never reported.

In addition to inadequate event classification, the MAUDE database also contains multiple types of incomplete or inaccurate classifications that go beyond the event field.

### Severity of risk unclear or unknown

There are no indicators beyond Event Type in the MAUDE database that precisely define the severity of reported device events. The current database only allows an assessment of the event’s outcome but, as noted above, it remains unclear whether and to what extent the event is related to the specific AI/ML device. While qualitative analysis of the report narratives is imaginable, such a project would require the use of fit-for-purpose text analysis and is not currently feasible on a large scale. As such, a data-based assessment of overall safety remains difficult to implement.

Moreover, the frequency of MDRs associated with a specific medical AI/ML device is not necessarily an accurate indicator of the level of risk or device failure. One might suspect that more reports imply a more problematic device, but that is not always the case. For example, DarioHealth’s Dario Blood Glucose Monitoring System (i.e., NBW) reports a total of 275 adverse events, according to Table 1. 231 of these were labeled as Malfunction but many were attributed to user error rather than actual device failure. Specifically, in one of the NBW malfunction reports, the user mentioned that he used an expired glucose strip cartridge, which led to a false negative result^26^. Another report similarly states that “the user realized that it was his own mistake ……. [t]herefore, it can be determined that there was misuse of the device.”^27^ For our purposes, these additional examples of user error suggest that there is not always a strong positive relationship between the numerosity of reports and the risk associated with a device. Separately, but relatedly, the salience of adverse events may differ across device types and user profiles, both of which might drive differences in the propensity to report problems in the first place. Finally, without an overall “denominator” for the frequency of a medical device’s use, it is not possible to talk about the relative safety of one product versus another^28^. This challenge also exists for other medical devices, but the interaction of known AI/ML capabilities with heterogeneous user profiles may lead to differential non-representativeness of MDRs for these products.

As a corollary, a smaller number of reports (or their absence) does not always imply lower product risk or severity. The WAVE Clinical Platform’s heart rate monitors (coded as MWI) are used by healthcare professionals to monitor patients’ waveforms, alarms, and results in real-time remotely. According to Table 1, MWI has only two event reports, which were marked as Malfunction. In both reports, the users noted that the MWI system failed to send emergency alerts to the intended recipients. Although these incidents did not result in severe patient outcomes, the examples emphasize that a recurrence of these incidents could result in serious injury or death. Again, our intention here is not to evaluate the risk or safety of specific devices per se, but rather to point out why and how it might be difficult for regulators and researchers to detect device-related safety issues by quantitatively analyzing the database’s indicators without reading through specific report narratives and in the absence of technology-appropriate categories for reporting problems.

### Problems without malfunctions are not tracked

According to the FDA, a product malfunction is an MDR reportable event if it results in the failure of the device to perform as intended in a way which could cause or contribute to a death or serious injury^7^. There are a few other triggering conditions but in general they are tied to a device’s contribution to death or serious injury. But many–perhaps most–problems that occur with AI/ML devices will not rise to this level of individual injury–either in practice or as a possibility. Indeed, many problems caused by medical AI/ML devices may not even occur as a result of malfunction, in the ordinary sense of that word. For example, suppose that a device predicts an 80% chance of a certain disease for a given patient. If the patient does not actually have the disease, is that a malfunction? Such a question is not possible to answer at an individual patient level. After all, an 80% chance of disease implies a 20% chance of its absence.

The only way to identify probabilistic reports as malfunctional (or not) is to look at performance across a large group of patients. For example, if we have 100 patients, and 30 of them have a certain disease, but the device produces an 80% probability for every single one of them, *then* one might say the device is malfunctioning—it appears miscalibrated. But this kind of aggregate-level malfunction would not make its way into local/individual level adverse event reports. The adverse event reporting system is designed at a patient/case level to identify local/individual issues, but many problems associated with AI/ML devices will present only at an aggregate or global level—i.e., they can be identified only by analyzing a large number of patient data points and comparing algorithmic performance to “true” diagnoses, outcomes, or base rates. Meanwhile, even if we could observe sub-population performance, a further problem would be non-random differences in the distributions of device errors—for example, error rates may differ across age, gender, ethnicity, race, etc^29^. This is likewise not something that can be tracked or ascertained from the existing MAUDE reports or MDR categories, but represents a known performance challenge for AI/ML devices. While this is not necessarily a defect of the MAUDE database—the MDR system was not originally designed to track such problems—it does point to the need for rethinking how we should approach postmarket surveillance of AI/ML devices, which we now turn to.

## Discussion

There are two potential ways to move forward and improve the current approach. The first is to consider how to improve the existing MAUDE database by reporting on characteristics that are particularly salient to AI/ML medical device safety (i.e., improve reporting characteristics). This includes reporting that goes beyond events which are traditionally considered as adverse events for ordinary (non AI/ML) medical devices. This is particularly important because AI/ML devices continue to evolve after they are brought to market^9^. This shift in emphasis toward post-market modifications could increase the burden on users to identify adverse events unless there is an improvement in the reporting system. To improve the reporting system, regulators should go beyond problems traditionally recognized as adverse events and take a more proactive role in ensuring device safety and usability. The second is to move beyond reporting altogether and consider a different post-market regulatory scheme for AI/ML devices. With respect to the first option, we make several suggestions about how to improve reporting. With respect to the second option, we do not seek to fully flesh out what substantial regulatory change might look like; rather, we limit ourselves to a few suggestions for what a new post-market regime could include.

We think the current reporting system could be reformed to enable analysis of three particularly important issues for evaluating the performance of AI/ML devices as described in^14^: concept drift, covariate shift, and algorithmic stability. These three concepts are at the root of what leads to the kinds of problems which we characterized above, including transferability, domain adaptation, cross-dataset evaluation, and subgroup discrepancies.

Concept drift refers to a situation in which the true joint feature/label distribution—the distribution that the algorithm is trying to approximate—changes slightly over time^9,10^. Another way to put it is that the relationship between inputs and outputs changes as the AI/ML system is deployed. The problem is well-known in the machine learning literature, and it is particularly salient in healthcare where we have massive amounts of data arriving continuously. For example, an AI/ML-based admission triage tool may be tested on a sample corresponding to a general healthcare environment, but then deployed in managing ICU queues. Or it may even be developed for an ICU unit in one sub-population, but then be deployed in a different sub-population, where the relationship between patient characteristics and health outcomes is not the same. For instance, by some measures the most prevalent chronic conditions for adults in New York State are high cholesterol and hypertension whereas in California they are obesity and heart disease. A tool that is initially deployed in New York but then adapted to California may be subject to concept drift. There is currently no means by which such issues could be reported in FDA’s system as they do not constitute an adverse event for reporting purposes. This is a problem that is distinctive, if not unique, to AI/ML medical devices.

How might the system do better at collecting data relevant to this issue? Reporting requirements could be modified so as to require, for instance, that for every AI/ML medical device, manufacturers must flag both when training data is significantly updated and when deployment conditions are substantially amended. There is of course some vagueness in what constitutes a substantial amendment, but as with current reporting practices, manufacturers could err on the safe side (i.e., in favor of being over inclusive in their reports). How often should such reports be made to regulators? While this is flexible, the FDA is currently updating MAUDE every three months and this could be a reasonable timeline: that is, manufacturers could submit a deployment conditions update quarterly, if changes are made within the previous three months. A further question is who should predefine benchmarks and performance metrics, the manufacturer or the FDA? We would encourage the FDA to issue recommendations to industry in the form of a draft guidance and seek their input; as they have done in defining performance and transparency metrics in the context of the total product life cycle (TPLC) approach (48).

Covariate shift refers to a situation in which the feature distribution alone changes^11^. This could occur if the training or early use data was not representative^1,30^, but it could happen for other reasons as well. As an extreme case, consider a situation where an AI/ML medical device such as Dario Health’s Dario Blood Glucose Monitoring System is trained on a sample of (high-risk for diabetes) obese younger men and then applied to a sample of (high risk for diabetes) non-obese older men. The distribution of diabetes among both groups could well be the same (i.e., the label distribution does not change) but the feature distribution is very different. Where this occurs, it can cause the performance of the algorithmic system to deteriorate gradually unless the manufacturer identifies the change and adapts to it. As above, there is currently no way to report such a phenomenon in the MAUDE database—the change in the feature distribution with respect to correlates of diabetes is not an “adverse event”. But the MAUDE database could be modified so as to encourage reports of issues encountered during such adaptive changes, so that hospitals, physicians, patients and other stakeholders are aware of how and where the device was initially developed and what genealogical changes relevant to its safety have since occurred. Further, such reports could help manufacturers improve labeling information and use/deployment training and education. To implement these changes, the same scheme as described above could be implemented—i.e., every three months a deployment conditions report is submitted if new information becomes available in the preceding quarter—indeed, the manufacturer could address both concept drift and covariate shift in one report. It is also worth noting that the feature distribution could change without manufacturers’ knowledge of it; while this is inevitable, the best we can do is to encourage reporting of known changes and likewise to encourage monitoring and investigation to quickly identify changes.

Another important property for AI/ML medical devices to satisfy is to produce similar outputs for similarly situated patients—we refer to this property as algorithmic stability^14^. This is similar to a “treat like cases alike” desideratum in the law, whereas in computer science it is referred to as the Lipschitz Property, which technically requires that for a given distance between two observations in the feature space, their distance should be similarly bounded in the output space. This sort of stability or robustness is especially important for AI/ML medical devices because we expect patients with similar characteristics to receive similar treatments of diagnoses. For example, consider a situation where a skin cancer screening device is implemented through a deep learning model which focuses on areas of large contract between a mole and the surrounding skin—so that paler patients are far more likely to have benign moles falsely classified as malignant. If we assume that the model has latched onto a property which is not medically more indicative of skin cancer, then two patients who are very similar in all the medically relevant characteristics can get very different skin cancer diagnoses. This is an instance of well-known adversarial robustness problems, but we have applied it to the medical context in particular.

Likewise, algorithmic stability can reveal itself in subgroup problems—the same kind of example that we gave above could give rise to racial differences in the algorithm’s performance which do not correspond to any underlying meaningful medical differences. Of course, sometimes there can be differences in disease distribution across racial categories, but algorithmic instability occurs where performance characteristics vary by race even in cases where the disease distribution is identical across race. Reporting on algorithmic stability will require requirements that are a little bit different from concept drift and covariate shift, as described above, because this is something that can happen at *patient* level, rather than at *population* level. However, in order to identify algorithmic instability, we need at a minimum a pairwise comparison of two similar patients. This is not something that can be identified for one patient alone—hence it is again not a “traditionally reportable” adverse event. Nonetheless, with a small change in reporting requirements, as suggested above, whereby manufacturers are required to submit a quarterly performance update, such instabilities can be identified by encouraging manufacturers to check explicitly for instability, including by making some pairwise comparisons of patients, as well as attempting to identify vulnerabilities—through, for example, auditing methods such as seeking out adversarial attacks^14^—and this can potentially be incorporated into subsequent device labelling and training information.

Beyond the problems described above, there are a number of other data reporting quality improvements that could be made if regulators like the FDA decide to adopt our approach of requiring periodic updates. Most of these reflect problems which have been discussed above. To address the extent of missing data, regulators may want to push for better quality control of the reports. Inadequate event classifications can be almost entirely eliminated in this way. Incomplete and inaccurate classifications can be more difficult to deal with; however, they can be minimized through better oversight and due diligence. Finally, disparities in subgroup performance, previously recognized as underreported^1^, can in part be tackled by requesting assessments of model performance across different demographic and socioeconomic characteristics.

One particularly interesting issue, however, is the role of human error. Our qualitative analysis of the adverse event reports highlights that many reports which are coded as algorithmic malfunctions were actually technician or analyst errors. However, the current adverse event reporting database does not track the distribution of analyst or technician errors, nor does it attempt to track the relationship between human factors and the AI/ML device. For example, suppose we have a diagnostic system for predicting diabetes risk, which outputs the log odds that a patient is at high risk for diabetes. This is fairly standard, since for example within a logistic model the log odds are a linear function of the predictors. However, most people will have trouble translating log odds to a probability. This could cause frequent errors in the diagnosis of diabetes even though the algorithm is not malfunctioning and there is no adverse event to be reported, even if we understand that term broadly. This problem highlights the fact that some issues which arise in the operation of AI/ML devices, including those discussed above, may be difficult to adequately address in the current model of database reporting even with changes. And that points to another possible way forward—namely, a more substantial revision of the regulatory approach, which we turn to next.

Thus far, we have discussed how to improve reporting and analysis of AI/ML errors within the existing MAUDE database framework. A more radical change would be to move beyond database-centric reporting. Since the problems associated with AI/ML devices can be quite difficult to capture in static adverse event reports, as described above, regulators may want to consider a different public health governance regime altogether. For example, Gerke argues in favor of “nutrition label” style reporting for AI/ML medical devices^31^. This is a valuable suggestion. Indeed, the Office of the National Coordinator for Health Information Technology (ONC) within the U.S. Department of Health and Human Services (HHS) has recently finalized a rule for predictive decision support interventions that are part of certified health, which would require such nutrition label style “model cards.”^32^ FDA could consider adopting this approach for AI/ML devices. Nutrition style labeling could help disentangle analyst error from device error; and such labels could disclose which sub-populations the AI/ML tool was trained and tested on. Labeling can inform analysts how to use the device, and hence reduce analyst error massively (note that analyst error is a frequent cause of adverse event reports in the MAUDE database). Moreover, labeling can disclose the demographic breakdowns of the testing population characteristics. Currently, most legally marketed devices do not disclose such characteristics^1^. Indeed, many authors have called for increased dataset diversity^33^ and such a regulatory approach would “build in” a mechanism for transparent reporting.

While a label cannot, by hypothesis, report when an evolutionary feature such as concept drift has occurred, it can warn users of the device about the possibility of concept drift when they apply the device in a new environment. For example: if the label makes it clear that a diagnostic device was tested on caucasian men between 35 and 49 years of age living in New York, then one can surmise that applying it to young black women in California could lead to deteriorated performance.

While nutrition style labels can be quite helpful, they can function even better if they are integrated into an environment of ongoing/regular reporting, as described in Ref. ^34^. Their system view approach envisions a cooperative and continuous reporting regime wherein the device manufacturers, users (i.e., hospitals and physicians), as well as policymakers treat the AI/ML device not as an isolated tool, but rather as one part of a larger care environment. This approach can encourage constructive dialogue: manufacturers can provide continuous updates; physicians may undergo regular training; and regulators may update both database disclosures and nutrition style labels. Indeed, the FDA’s proposed Total Product Lifecycle Regulatory Approach is developed in this spirit^35,36^.

## Methods

We collected data from the FDA’s medical device adverse event reporting database (the “Manufacturer and User Facility Device Experience” or MAUDE database). Data were gathered from FDA’s downloadable 510(k) files^37^, restricting to medical devices approved from 2010 through 2023, inclusive. This includes both Class I and II Premarket Notification (“510(k)”) devices and De Novo classification requests for low to moderate risk medical devices. These devices represent over 98% of all FDA device market authorizations over the same period and all but two of the 882 devices that the FDA had classified as AI/ML devices over the period of study.

We calculated the number of adverse events within pre-defined timeframes post-approval (3, 6, 9, 12, 24 months) using the FDA’s Medical Device Reporting (MDR) system^37^, and flagging mandatory (i.e., more serious) adverse event reports. Additionally, we created a flag for all devices included in the FDA’s Artificial Intelligence and Machine Learning (AI/ML)-Enabled Medical Devices list^38^. Using NyquistAI’s database, we also flagged all devices that contained the term “software” in their device ID, device name, indications for use, or device summary file in order to establish a relevant set of digital/digitally-enabled devices. Our final dataset comprises 823 unique 510(k)-cleared devices that could be linked to a total of 943 subsequent adverse events reported (MDRs for short) between 2010 and 2023. This dataset tracks 54 features related to the reported events and device manufacturers. These features include the type of event, the setting where it occurred, the associated manufacturer and product, and so forth. Within the 943 linked MDRs, there are 20 unique medical device product codes (i.e., unique types of medical devices). A product code is a three-letter identifier assigned by the FDA’s Center for Devices and Radiological Health (CDRH) or certain devices regulated by the Center for Biologics Evaluation and Research (CBER). This code serves to internally classify and track medical devices by linking a device type with its designated product classification for a specific application. These codes, along with device names and attributes, are assigned by CDRH to facilitate regulatory processes. We have provided links to both our complete dataset and the associated STATA code for reproducing the full analysis at the end of this manuscript.

## Conclusion

In sum, this study closely considers the FDA’s MAUDE database, focusing in particular on adverse event reports associated with AI/ML-based medical devices that received marketing authorization from 2010 through 2023. We find the MAUDE database to be significantly lacking in its informational value: among the features chosen for reporting, there is substantial missing data (some columns are missing entirely). For variables that are not missing, the information included is often inaccurate, vague, or misleading. Meanwhile, the most significant risks associated with AI/ML devices—for example, those stemming from the nature, size, location, and representativeness of the models’ training and validation data—are not reported at all. We have described these problems and made two sets of suggestions. The first contains recommendations for improving the relevance of the MAUDE database for AI/ML devices. The second includes recommendations for moving beyond the MAUDE database and reimagining transparent post-market surveillance of AI/ML products in the absence of individual event-based reporting.

## Supplementary information

Supplementary Information

## Data Availability

The datasets analyzed in this study are publicly available at https://github.com/melificient/medAI. The repository includes two Stata 18 datasets, created on January 29, 2025: 823_fda_devices_withAEs.dta: A dataset of 823 unique 510(k)-cleared medical devices approved by the FDA between 2010 and 2023, identified as incorporating Artificial Intelligence and/or Machine Learning technologies. 943_AEs_for_fda_devices.dta: A corresponding dataset of 943 adverse event reports (Medical Device Reports, or MDRs) for the above devices, covering the same time period. There are no restrictions on the use of these datasets.

## Code availability

All custom code used for dataset creation, cleaning, and analysis was developed in Stata 18 and is publicly available at https://github.com/melificient/medAI. The primary script, create_MedAI_datasets.do, relies on input files located in the raw/ subdirectory. No proprietary code was used. The provided do file enables full reproduction of the datasets generation. There are no access restrictions to the code.

## References

[1] Muralidharan, V. et al. A scoping review of reporting gaps in FDA-approved AI medical devices. *npj Digit. Med.* **7**, 273 (2024). 39362934 10.1038/s41746-024-01270-x PMC11450195

[2] Mashar, M. et al. Artificial intelligence algorithms in health care: Is the current food and drug administration regulation sufficient?. *JMIR AI* **2**, e42940 (2023). 38875544 10.2196/42940 PMC11041443

[3] Wu, E. et al. Toward stronger FDA approval standards for AI medical devices. *Stanford HAI Policy Brief* 1–6 (2022).

[4] U.S. Food and Drug Administration. *November**20*–*21*, *2024*: *Digital Health Advisory Committee meeting*. https://www.fda.gov/advisory-committees/advisory-committee-calendar/november-20-21-2024-digital-health-advisory-committee-meeting-announcement-11202024

[5] U.S. Food and Drug Administration. Artificial intelligence and machine learning (AI/ML)-enabled medical devices (accessed June https://www.fda.gov/medical-devices/software-medical-device-samd/artificial-intelligence-and-machine-learning-aiml-enabled-medical-devices 2024).

[6] U.S. Food and Drug Administration. A history of medical device regulation and oversight in the US (2023). https://www.fda.gov/medical-devices/overview-device-regulation/history-medical-device-regulation-oversight-united-states

[7] U.S. Food and Drug Administration. *Medical device reporting for manufacturers: guidance for industry and food and drug administration staff* https://www.fda.gov/media/86420/download (2016).

[8] Stern, A. D. The regulation of medical AI: Policy approaches, data, and innovation incentives. *Natl. Bur. Econ. Res*. *Working Paper**30639*10.3386/w30639 (2022).

[9] Babic, B., Gerke, S., Evgeniou, T. & Cohen, I. G. Algorithms on regulatory lockdown in medicine. *Science* **366**, 1202–1204 (2019). 31806804 10.1126/science.aay9547

[10] Gama, J., Žliobaitė, I., Bifet, A., Pechenizkiy, M. & Bouchachia, A. A survey on concept drift adaptation. *ACM Comput. Surv.* **46**, 1–37 (2014).

[11] Bickel, S., Brückner, M. & Scheffer, T. Discriminative learning for differing training and test distributions. *Proc. 24th Int. Conf. Mach. Learn. (ICML)* **81**, 88 (2007).

[12] Webb, G. I. et al. Characterizing concept drift. *Data Min. Knowl. Disc.* **30**, 964–994 (2016).

[13] Lu, J. et al. Learning under concept drift: A review. *IEEE Trans. Knowl. Data Eng.* **31**, 2346–2363 (2019).

[14] Goodfellow, I. J., Shlens, J. & Szegedy, C. Explaining and harnessing adversarial examples. *In Proceedings of the 3rd International Conference on Learning Representations (ICLR)*, (2015).

[15] Garcelon, N., Burgun, A., Salomon, R. & Neuraz, A. Electronic health records for the diagnosis of rare diseases. *Kidney Int.* **97**, 676–686 (2020). 32111372 10.1016/j.kint.2019.11.037

[16] Abràmoff, M. D. et al. Considerations for addressing bias in artificial intelligence for health equity. *npj Digit. Med.* **6**, 170 (2023). 37700029 10.1038/s41746-023-00913-9 PMC10497548

[17] Lyell, D., Wang, Y., Coiera, E. & Magrabi, F. More than algorithms: an analysis of safety events involving ML-enabled medical devices reported to the FDA. *J. Am. Med. Inform. Assoc.* **30**, 1227–1236 (2023). 37071804 10.1093/jamia/ocad065 PMC10280342

[18] Ross, C. As the FDA clears a flood of AI tools, missing data raise troubling questions on safety and fairness. *STAT* https://www.statnews.com/2021/02/03/fda-clearances-artificial-intelligence-data/ (2023).

[19] U. S. Food & Drug Administration. MAUDE Adverse Event Report System: MDRFOI ID 16156042 (accessed June 2024). https://www.accessdata.fda.gov/scripts/cdrh/cfdocs/cfmaude/detail.cfm?mdrfoi__id=16156042

[20] U.S. Food & Drug Administration. MAUDE Adverse Event Report System: MDRFOI ID 9068969 (accessed June 2024). https://www.accessdata.fda.gov/scripts/cdrh/cfdocs/cfmaude/detail.cfm?mdrfoi__id=9068969

[21] U.S. Food & Drug Administration. MAUDE Adverse Event Report System: MDRFOI ID 8193108 (accessed June 2024). https://www.accessdata.fda.gov/scripts/cdrh/cfdocs/cfmaude/detail.cfm?mdrfoi__id=8193108

[22] U.S. Food & Drug Administration. MAUDE Adverse Event Report System: MDRFOI ID 8389338 (accessed June 2024). https://www.accessdata.fda.gov/scripts/cdrh/cfdocs/cfmaude/detail.cfm?mdrfoi__id=8389338

[23] U.S. Food & Drug Administration. MAUDE Adverse Event Report System: MDRFOI ID 8461676 (accessed June 2024).https://www.accessdata.fda.gov/scripts/cdrh/cfdocs/cfmaude/detail.cfm?mdrfoi__id=8461676

[24] Fogliato, R., Xiang, A., Lipton, Z., Nagin, D. & Chouldechova, A. On the validity of arrest as a proxy for offense: Race and the likelihood of arrest for violent crimes. *Proc. AAAI/ACM Conf*. *AI Ethics Soc*. **34**, 100–111 (2021).

[25] Fogliato, R., Chouldechova, A. & G’Sell, M. Fairness evaluation in presence of biased noisy labels. *Proc. Twenty Third Int. Conf. Artif. Intell. Stat. PMLR* **108**, 2325–2336 (2020).

[26] U.S. Food & Drug Administration. MAUDE Adverse Event Report System: MDRFOI ID 12328586 (accessed June 2024). https://www.accessdata.fda.gov/scripts/cdrh/cfdocs/cfmaude/detail.cfm?mdrfoi__id=12328586

[27] U.S. Food & Drug Administration. MAUDE Adverse Event Report System: MDRFOI ID 10114222 (accessed June 2024). https://www.accessdata.fda.gov/scripts/cdrh/cfdocs/cfmaude/detail.cfm?mdrfoi__id=10114222

[28] Chandra, A., Kao, J., Miller, K. L. & Stern, A. D. Regulatory incentives for innovation: The FDA’s breakthrough therapy designation. *Rev. Econ. Stat.* **106**, 1–46 (2024).

[29] Barwise, A. et al. What contributes to diagnostic error or delay? A qualitative exploration across diverse acute care settings in the United States. *J. Patient Saf.* **17**, 239–248 (2021). 33852544 10.1097/PTS.0000000000000817 PMC8195035

[30] Fox-Rawlings, S. R., Gottschalk, L. B., Doamekpor, L. A. & Zuckerman, D. M. Diversity in medical device clinical trials: do we know what works for which patients?. *Milbank Q* **96**, 499–529 (2018). 30203600 10.1111/1468-0009.12344 PMC6131322

[31] Gerke, S. Nutrition facts labels’ for artificial intelligence/machine learning-based medical devices—the urgent need for labeling standards. *George Wash. Law Rev.* **91**, 79 (2023).

[32] Health data, technology, and interoperability: Certification program updates, algorithm transparency, and information sharing, 89 FR 1192 https://www.federalregister.gov/documents/2024/01/09/2023-28857/health-data-technology-and-interoperability-certification-program-updates-algorithm-transparency-and (2024).

[33] Arora, A. et al. The value of standards for health datasets in artificial intelligence-based applications. *Nat. Med.* **29**, 2929–2938 (2023). 37884627 10.1038/s41591-023-02608-w PMC10667100

[34] Gerke, S. et al. The need for a system view to regulate artificial intelligence/machine learning-based software as medical device. *npj Digit. Med.* **3**, 53 (2020). 32285013 10.1038/s41746-020-0262-2 PMC7138819

[35] U.S. Food and Drug Administration. Proposed regulatory framework for modifications to artificial intelligence/machine learning (AI/ML)-based software as a medical device (SaMD): discussion paper and request for feedback https://www.fda.gov/media/122535/download (2019).

[36] Center for devices and radiological health. Total product life cycle for medical devices. U.S. Food and Drug Administration https://www.fda.gov/about-fda/cdrh-transparency/total-product-life-cycle-medical-devices (2023).

[37] U.S. Food and Drug Administration. *MDR data files* (accessed January 5, 2024). https://www.fda.gov/medical-devices/medical-device-reporting-mdr-how-report-medical-device-problems/mdr-data-files

[38] U.S. Food and Drug Administration. *Artificial intelligence and machine learning (AI/ML)-enabled medical devices* (last updated May 13, 2024). https://www.fda.gov/medical-devices/software-medical-device-samd/artificial-intelligence-and-machine-learning-aiml-enabled-medical-devices

