# Supplementary material for: A general framework for governing marketed AI/ML medical devices
Source: NPJ Digit Med. 2025 May 31;8:328. doi: 10.1038/s41746-025-01717-9 (PMC12126487; doi:10.1038/s41746-025-01717-9)
Supplement: Supplementary file 1 — Supplementary Information [file 41746_2025_1717_MOESM1_ESM.docx]

# **Supplementary information**

**
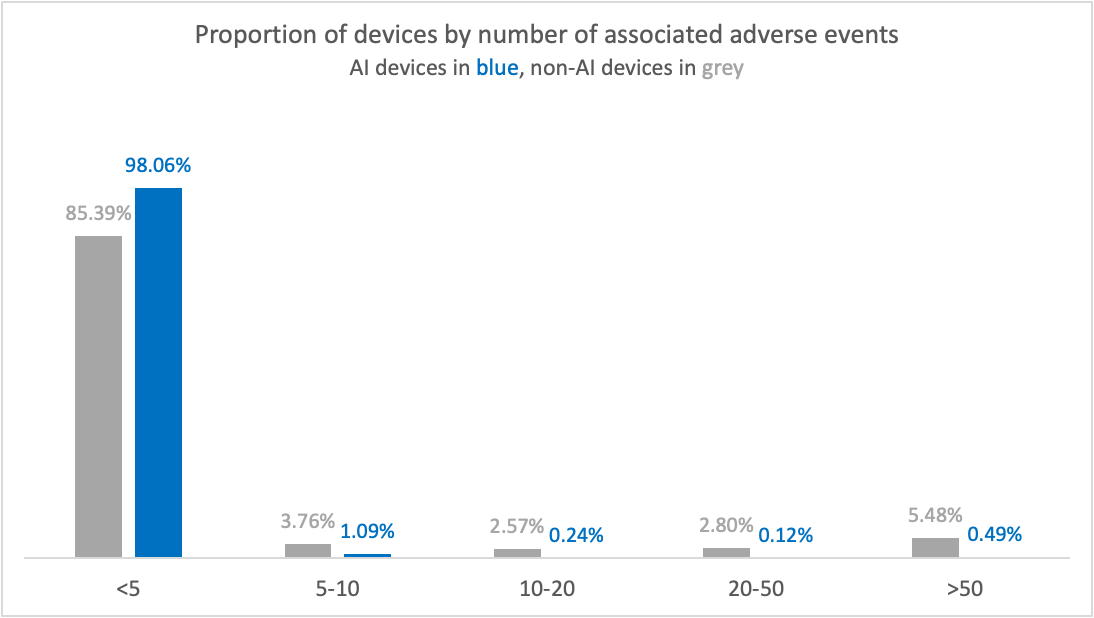
**

**Supplementary Figure 1. Share of devices categorized by adverse event count.**

The plot illustrates the extent of concentration of adverse events across devices, showing that over 98% of adverse events associated with AI/ML devices are linked to fewer than five devices. While non-AI/ML devices also show concentration, only about 85% fall within the same range, indicating a comparatively more dispersed pattern of adverse event reporting.

**
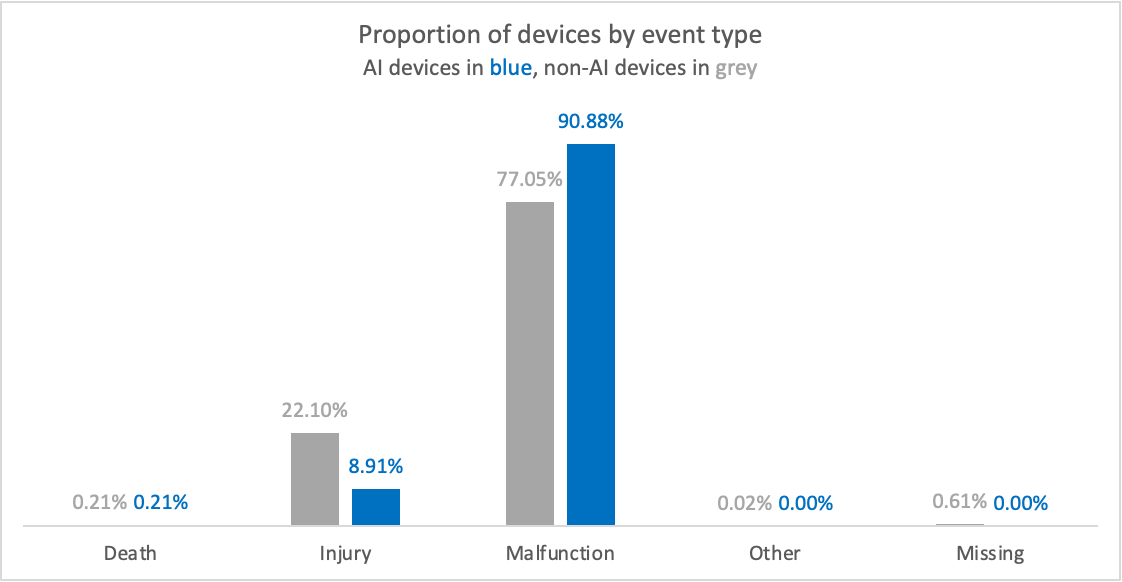
**

**Supplementary Figure 2. Proportion of devices distributed across adverse event types**

The figure represents the distribution of reported adverse event types—malfunction, injury, and others—for both AI/ML-enabled and non-AI/ML medical devices. While malfunctions dominate in both categories, AI/ML devices exhibit a notably higher concentration, with injuries accounting for fewer than 10% of events, compared to over 20% in non-AI/ML devices.
